# Acute corticospinal tract diffusion tensor imaging predicts 6-month functional outcome after intracerebral haemorrhage

**DOI:** 10.1007/s00415-022-11245-1

**Published:** 2022-07-21

**Authors:** G. Schwarz, B. Kanber, F. Prados, S. Browning, R. Simister, R. Jäger, G. Ambler, C. A. M. Gandini Wheeler-Kingshott, David J. Werring

**Affiliations:** 1 Neurologia, Stroke Unit, ASST Grande Ospedale Metropolitano Niguarda, Milan, Italy; 2 Department of Brain Repair and Rehabilitation, Stroke Research Centre, UCL Queen Square Institute of Neurology, National Hospital for Neurology and Neurosurgery, University College London, Queen Square, London, WC1N UK; 3 NMR Research Unit, Department of Neuroinflammation, Faculty of Brain Sciences, Queen Square Multiple Sclerosis Centre, Queen Square Institute of Neurology, University College London (UCL), London, UK; 4 Department of Medical Physics and Biomedical Engineering, Centre for Medical Image Computing, UCL, London, UK; 5 National Institute for Health Research, Biomedical Research Centre, University College London Hospitals, London, UK; 6 e-Health Center, Universitat Oberta de Catalunya, Barcelona, Spain; 7 Department of Statistical Science, University College London, Gower Street, London, UK; 8 Lysholm Department of Neuroradiology and the Neuroradiological Academic Unit, Department of Brain Repair and Rehabilitation, UCL Institute of Neurology, Queen Square, London, UK; 9 Department of Brain and Behavioural Sciences, University of Pavia, Pavia, Italy; 10 Brain Connectivity Center, IRCCS Mondino Foundation, Pavia, Italy

**Keywords:** Deep intracerebral haemorrhage, Outcome prediction, Fractional anisotropy, Mean diffusivity, ICH score, Corticospinal tract

## Abstract

**Introduction:**

Diffusion tensor imaging (DTI) can assess the structural integrity of the corticospinal tract (CST) in vivo. We aimed to investigate whether CST DTI metrics after intracerebral haemorrhage (ICH) are associated with 6-month functional outcome and can improve the predictive performance of the existing ICH score.

**Methods:**

We retrospectively included 42 patients with DTI performed within 5 days after deep supratentorial spontaneous ICH. Ipsilesional-to-contralesional ratios were calculated for fractional anisotropy (rFA) and mean diffusivity (rMD) in the pontine segment (PS) of the CST. We determined the most predictive variables for poor 6-month functional outcome [modified Rankin Scale (mRS) > 2] using the least absolute shrinkage and selection operator (LASSO) method. We calculated discrimination using optimism-adjusted estimation of the area under the curve (AUC).

**Results:**

Patients with 6-month mRS > 2 had lower rFA (0.945 [± 0.139] vs 1.045 [± 0.130]; OR 0.004 [95% CI 0.00–0.77]; *p* =  0.04) and higher rMD (1.233 [± 0.418] vs 0.963 [± 0.211]; OR 22.5 [95% CI 1.46–519.68]; *p* = 0.02). Discrimination (AUC) values were: 0.76 (95% CI 0.61–0.91) for the ICH score, 0.71 (95% CI 0.54–0.89) for rFA, and 0.72 (95% CI 0.61–0.91) for rMD. Combined models with DTI and non-DTI variables offer an improvement in discrimination: for the best model, the AUC was 0.82 ([95% CI 0.68–0.95]; *p* = 0.15).

**Conclusion:**

In our exploratory study, PS-CST rFA and rMD had comparable predictive ability to the ICH score for 6-month functional outcome. Adding DTI metrics to clinical-radiological scores might improve discrimination, but this needs to be investigated in larger studies.

## Introduction

Spontaneous (non-traumatic) intracerebral haemorrhage (ICH) is a severe form of stroke, accounting for nearly 3 million deaths globally in 2017 [1]. About 40% of patients die within the first month, while about 80% of those who survive are functionally dependent on others [2]. Given the frequently poor clinical outcome after ICH, clinicians are often faced with difficult decisions regarding the appropriate level of acute treatment, for example admission to critical care or neurosurgery. Early functional outcome prediction after ICH is thus fundamental in everyday clinical practice to guide subsequent care. Many clinical-radiological prognostic scores have been proposed, of which the ICH score is one of the most validated, being initially designed to predict 30-day mortality [3]. The ICH score ranges from 0 to 6, including simple clinical and radiological variables: GCS score (2 point if GCS 3 to 4 and 1 point if GCS score 5–12), age (1 point if ≥ 80 years), ICH site (1 point for infratentorial origin), ICH volume (1 point if ≥ 30 cm^3^) and the presence of intraventricular haemorrhage (1 point if present). The ICH score has modest discrimination performance for functional outcome (typical c-index around 0.70–0.80), as do other similar prognostic instruments [4]. Most available studies predict mortality at 30 days or 3-months functional outcome, with decreasing predictive performance as the time interval from the index event increases [4]. However, functional improvement after ICH can occur beyond 3-months [5, 6], and full recovery from intracerebral haemorrhage may take longer than that from acute ischaemic stroke; a reliable 6-month functional outcome prediction instrument would therefore be very useful for clinicians in the acute phase of ICH.

The modified Rankin Scale (mRS) [7]—a 6-point scale with possible disability scores ranging from 0 (no residual symptoms) to 5 (severe disability, bedridden, requires continuous care), and with score 6 for patients who have died—is strongly related to motor recovery which, in turn, is likely to be dependent on the integrity of the corticospinal tract (CST) [8, 9].

Diffusion tensor imaging (DTI), as a measure of brain microstructure, is thus a promising biomarker to predict long-term motor outcome after both ischaemic stroke [10] and ICH [11]. Based on fractional anisotropy (FA) and mean diffusivity (MD) changes, DTI provides quantitative in vivo information regarding the structural integrity of brain tissue including white matter tracts, even where these appear normal on standard structural MRI. Several studies have examined whether DTI-derived data, applied to assess the integrity of CST, can predict motor outcome after ICH. However, these studies were methodologically heterogeneous and focused on early (3 months) outcome and have provided inconsistent results. Furthermore, most studies did not investigate the added predictive value of DTI in addition to conventional clinical scores [11].

We therefore assessed the integrity of the pontine segment of CST (PS-CST) via DTI in the early phase (within 5 days) after symptom onset in patients with deep supratentorial ICH. We included only this ICH location because of its proximity and likely consistent impact on the fibres of the ipsilateral CST; by contrast, lobar ICH may have an erratic impact on CST, while infratentorial ICH may be located *within* the PS-CST. We aimed to investigate the predictive performance of early-phase PS-CST DTI metrics acquired in the first 5 days from ICH onset, both alone and when added to the ICH score.

## Methods

### Study population

We retrospectively included consecutive patients with first-ever deep supratentorial ICH (with no previous history of ICH or ischaemic stroke) from the prospective SIGNaL (Stroke InvestiGation in North and Central London) registry who presented from January 2017 to March 2019. Other inclusion criteria were: 6-month follow-up data available and DTI sequences (of adequate quality for analysis) acquired within 5 days after ICH onset. We excluded patients with secondary causes of ICH (tumour, vascular malformation, aneurysm, vasculitis, venous infarction or haemorrhagic transformation of an infarct), history of trauma, or ICH located in lobar or infratentorial regions.

### Clinical evaluation

We retrieved baseline detailed demographic, clinical and radiological information. Six-month functional outcome was assessed via mRS at follow-up visits or by phone call. Data were collected as part of routine clinical care, and data analysis was approved as a service evaluation by the University College London Hospital Trust Data Governance Review Board.

### MRI imaging acquisition

All MRI scans were performed on a Philips Achieva 3 Tesla scanner (Philips, Best, Netherlands). The following acquisitions were included: single-shell diffusion-weighted imaging (DWI) (voxel resolution 0.9 × 0.9 ×  5 mm^3^, echo time 76 ms, repetition time 3.5 s, flip angle 90°) comprising one *b* = 0, followed by six b = 1000 s/mm^2^ volumes; T1-weighted imaging (voxel resolution 0.94 × 0.94 × 1.1 mm^3^, echo time 3.3 ms, repetition time 7.1 ms, flip angle 9°); fluid-attenuated inversion recovery (FLAIR) imaging (voxel resolution 0.45 × 0.45 × 4 mm^3^, echo time 110 ms, repetition time 10.8 s, inversion time 2.8 s, flip angle 90°); and susceptibility-weighted imaging (SWI) (voxel resolution 0.24 × 0.24 × 1 mm^3^, repetition time 31 ms, flip angle 17°).

### MRI analysis and lesion segmentation

Every MRI was evaluated by a single rater (GS) blinded to other clinical variables. ICH and peri-haematomal oedema (PHE) regions were manually segmented on SWI and FLAIR sequences, respectively. The regions of interest (ROI) obtained from ICH and PHE segmentations were used to obtain the ICH and PHE volumes. ICH location was assessed using the Cerebral Haemorrhage Anatomical Rating Instrument (CHARTS) [12]. The ICH score was calculated for every patient according to the original publication [3].

### MRI image processing and DTI metrics

**Fig. 1:**
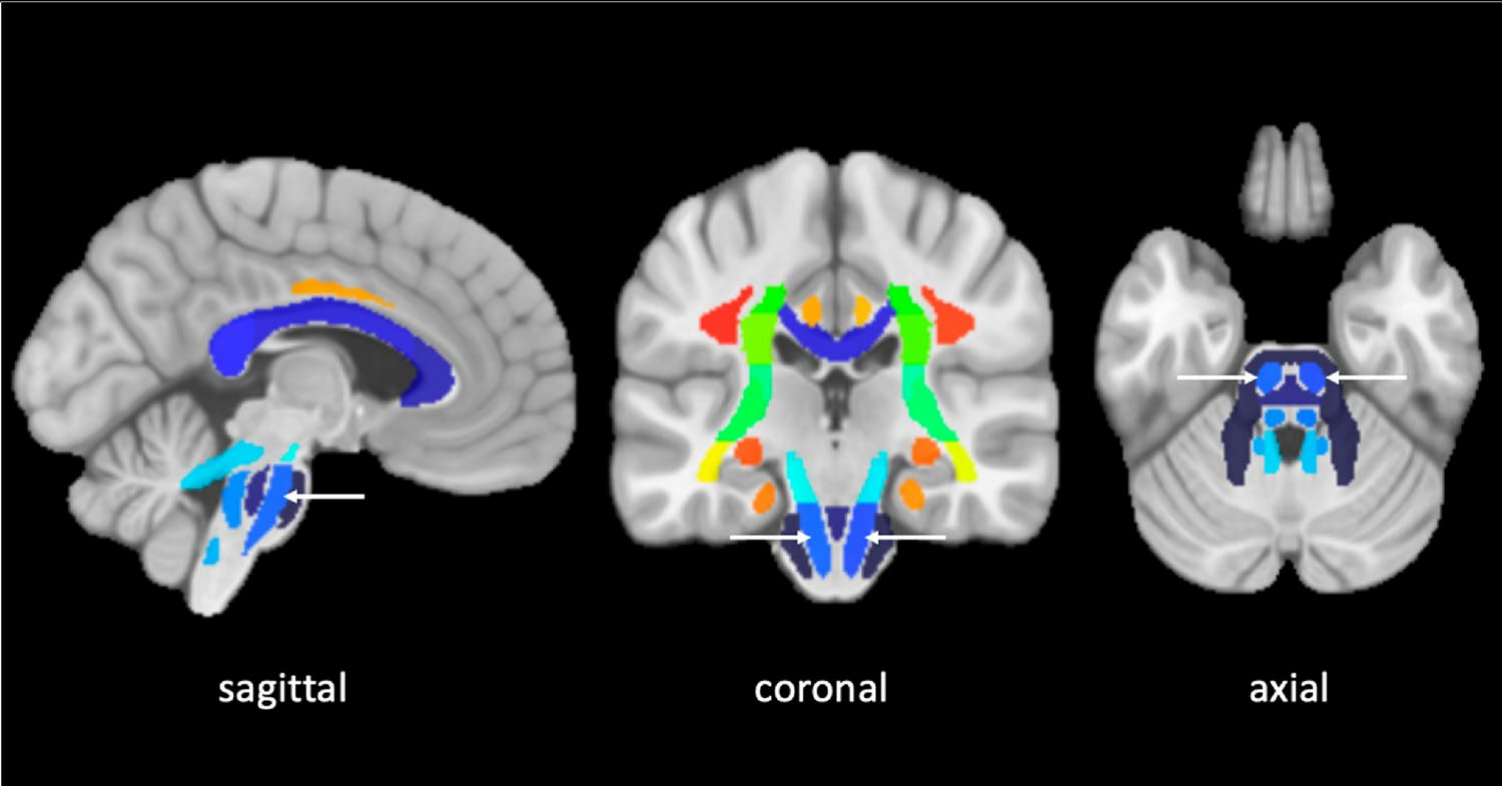
John Hopkins University [JHU] DTI-based white matter atlas: in blue the pontine segment of the corticospinal tract (PS-CST) (white arrow), on sagittal, coronal and axial plane

DWI data were corrected for eddy currents prior to DTI fitting using FSL [13]. ROIs for the CST and the PS-CST were obtained using the John Hopkins University [JHU] DTI-based white matter atlas (https://identifiers.org/neurovault.image:1401) (Fig. 1) non-rigidly transformed to each subject’s DWI image space. Similarly, to obtain the ICH and PHE ROIs in each subject’s DWI space, non-rigid transformations were computed between SWI and DWI, and between FLAIR and DWI image spaces. All image transformations were done using the NiftyReg software package [14]. Finally, mean FA and MD were computed in the CST and in the PS-CST using the obtained ROIs. To obtain the ICH probability map, we summed all the lesion masks and divided by the number of patients to give a lesion probability at each voxel.

### Statistical analysis

Ipsilesional-to-contralesional PS-CST ratios were calculated for FA (rFA = FA affected side/FA unaffected side) and MD (rMD = MD affected side / MD in unaffected side). Long-term (6 months) functional outcome was defined as *poor* if mRS was greater than 2. The association between clinical and radiological variables and poor 6-month functional outcome was assessed via univariable logistic regression analysis. rFA, rMD and ICH score discrimination for poor functional outcome was assessed by calculating the area under the ROC (receiver operating characteristic) curve (AUC). After selection of DTI (rFA and rMD) and non-DTI predictors (standard clinical and radiological variables) from univariable analyses (*p* < 0.10), we fitted logistic regression models using least absolute shrinkage and selection operator (LASSO) estimation [15] to find variables with best predictive ability for poor mRS at 6 months. Two different models were obtained: Model 1 combined DTI *plus* non-DTI variables, while Model 2 combined DTI *plus* the ICH score. We validated the models using bootstrapping and calculated optimism-adjusted estimates of the AUC [16]. We fitted both models (using standard logistic regression)-both including the ICH score, to obtain nested models - and compared the fits using the likelihood ratio (LR) test. Statistical analysis was performed using STATA 16 (StataCorp, College Station, TX). The statistical significance level was set at *p* = 0.05.

## Results

**Fig. 2:**
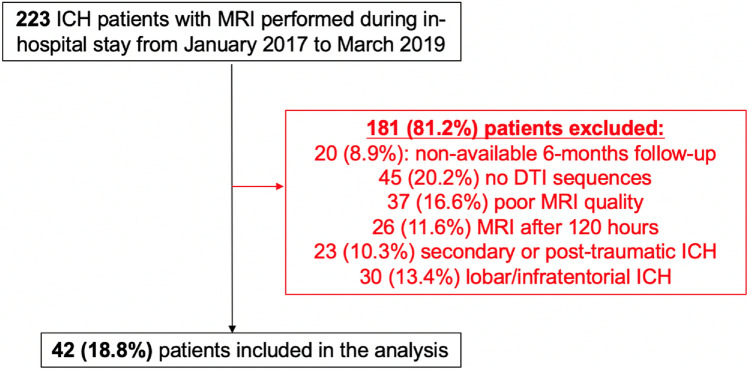
Flow-chart for patients included in the analysis

**Fig. 3:**
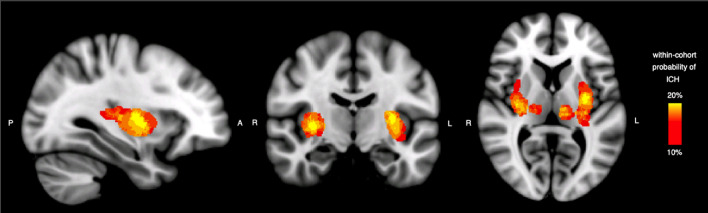
Within-cohort ICH probability map in red-to-yellow hotwire scale (lesion probability range 10–20%) overlaid on the 1 mm Montreal Neurological Institute (MNI) 152 anatomical template

We included 42 adult patients with spontaneous supratentorial deep ICH (flow chart in Fig. 2). Group probability maps showed the ICH lesion distribution reported in Fig. 3. Table 1 summarizes clinical and radiological characteristics in the entire cohort, including PS-CST mean rFA and rMD. Median age was 62 years (IQR 52–72), and median ICH volume was 5.4 mL (IQR 3.0–11.7). Thirteen patients (31.0%) had poor functional outcome (mRS > 2) at 6 months. Mean rFA was 1.024 (SD 0.139), and mean rMD was 1.046 (SD 0.312). Table 2 describes univariable logistic regression models for the association between clinical and radiological variables, including rFA and rMD, and poor long-term functional outcome.

**Table 1:** Clinical/radiological variables, FA and MD ratios in the corticospinal tract for the entire cohort

	Entire cohort *N* = 42 (%)
**General variables**	
Age	
Median (IQR)	62.0 (52.0–72.0)
< 80	36 (85.7)
≥ 80	6 (14.3)
GCS	
15	40 (95.2)
< 15	2 (4.8)
Gender	
Female	12 (28.6)
Male	30 (71.4)
mRS pre-ICH	
0–2	41 (97.6)
3–5	1 (2.4)
mRS at 6 months	
0	8 (19.0)
1	10 (23.8)
2	11 (26.2)
3	7 (16.7)
4	2 (4.8)
5	2 (4.8)
6	2 (4.8)
ICH score	
0	31 (73.8)
1	9 (21.4)
2	2 (4.8)
**Standard Neuroimaging Variables**	
ICH volume	
Median (IQR)	5.4 (3.0–11.7)
PHE volume	
Median (IQR)	12.6 (7.5–19.3)
Side of ICH	
Left	24 (57.1)
Right	18 (42.9)
IVH	
Yes	7 (17.7)
No	35 (83.3)
**DTI metrics**	
PS-CST rFA (Mean [SD])	1.014 [0.139]
PS-CST rMD (Mean [SD])	1.046 [0.312]

*ICH* intracerebral haemorrhage, *mRS* modified Rankin scale, *FA* fractional anisotropy, *MD* mean diffusivity, *PS-CST* pontine segment of corticospinal tract, *IVH* intra-ventricular haemorrhage, *PHE* peri-haematomal oedema, *GCS* Glasgow Coma Scale, *DTI* diffusion tensor imaging, *CST FA ratio* FA affected side/FA unaffected side, *CST MD ratio* MD affected side / MD unaffected side

**Table 2:** Univariable logistic regression models (with odds ratios) for the association between clinical/radiological variables and DTI metrics with poor functional outcome (mRS > 2) at 6 months

	mRS 0–2 *N* = 29 (%)	mRS 3–6 *N* = 13 (%)	OR (95% CI)	*P* value
**General variables**				
Age				
Median (IQR)	60.0 (49.0–68.0)	72.0 (62.0–80.0)	1.06 (1.00–1.12)^a^	0.039
GCS				
15	28 (96.5)	12 (92.3)	2.33 (0.13–40.46)	0.561
< 15	1 (3.5)	1 (7.7)		
Gender				
Female	7 (24.1)	5 (38.5)	0.51 (0.13–2.07)	0.346
Male	22 (75.9)	8 (61.5)		
ICH score^b^				
0	26 (89.7)	5 (38.5)	13.87 (2.70–71.2)	0.002
1	3 (10.3)	8 (61.5)		
**Standard neuroimaging variables**				
ICH volume				
Median (IQR)	5.1 (3.4–10.3)	6.3 (3.0–21.5)	1.25 (0.75–2.08)^c,d^	0.397
PHE volume				
Median (IQR)	12.6 (7.8–18.8)	13.1 (5.9–22.8)	0.83 (0.39–1.77)^c,d^	0.621
Side of ICH				
Left	17 (58.6)	7 (53.9)	1.21 (0.33–4.53)	0.773
Right	12 (41.4)	6 (46.1)		
IVH				
No	28 (96.5)	7 (53.9)	24.0 (2.47–233.06)	0.006
Yes	1 (3.5)	6 (46.1)		
**DTI metrics**				
PS-CST rFA (Mean [SD])	1.045 (0.130)	0.945 (0.139)	0.004 (0.00–0.77)	0.04
PS-CST rMD (Mean [SD])	0.963 (0.211)	1.233 (0.418)	22.5 (1.46–519.68)	0.02

*ICH* intracerebral haemorrhage, *mRS* modified Rankin scale, *FA* fractional anisotropy, *MD* mean diffusivity, *PS-CST* pontine segment of corticospinal tract, *IVH* intra-ventricular haemorrhage, *PHE* peri-haematomal oedema, *GCS* Glasgow Coma Scale, *DTI* diffusion tensor imaging

^a^per 1 year increase

^b^Only 2 patients scored 2 on ICH score: these 2 patients were added to ICH score 1

^c^per 1 ml increase

^d^Volumes were log-transformed

Patients with poor functional outcome were older (median age 72 [IQR 62–80] *vs* 60 [IQR 49–68]; OR per 1 year increase 1.06 [1.00–1.12]; *p* = 0.039) and had higher ICH scores (OR 13.87 for score 1 *vs* score 0 [95% CI 2.70–71.20]; *p* = 0.002). Intraventricular haemorrhage (IVH) was associated with poor functional outcome (OR 24.0 [95% CI 2.47–233.06]; *p* = 0.006). Patients with poor long-term mRS had significantly lower rFA (0.945 [± 0.139] *vs* 1.045 [± 0.130]; OR 0.004 [95% CI 0.00–0.77]; *p* = 0.04) and significantly higher rMD (1.233 [± 0.418] *vs* 0.963 [± 0.211]; OR 22.5 [95% CI 1.46–519.68]; *p* = 0.02) in the PS-CST.

The discrimination of the ICH score for poor functional outcome at 6-months, measured by the AUC, was 0.76 (95% CI 0.61–0.91); discrimination for rFA and rMD was 0.71 (95% CI 0.54–0.89) and 0.72 (95% CI 0.61–0.91), respectively.

We built two different LASSO regression models (Table 3). In Model 1, we included both DTI (rFA and rMD) and non-DTI metrics (age and IVH): all the variables included in the model were selected via LASSO regression analysis, and the optimism-adjusted predictive ability for poor mRS at 6 months was 0.81 (95% CI 0.69–0.94). In Model 2, we included both DTI variables (rFA and rMD) and ICH score; rMD and ICH score were selected, and the optimism-adjusted AUC for this model was 0.82 (95% CI 0.68–0.95). The LR test to evaluate the statistical significance of the difference in the predictive ability between Model 1 and Model 2 against the ICH score alone gave *p* values of 0.13 and 0.15, respectively.

**Table 3:** Long-term (6-month) poor (mRS > 2) functional outcome prediction using LASSO regression

	Variables included in the LASSO regression analysis	Variables selected via LASSO regression analysis	Optimism-adjusted AUC (95% CI) for the selected variables
**Model 1**	PS-CST rFA	PS-CST rFA	0.81 (0.69–0.94)
	PS-CST rMD	CST rMD	
	Age	Age	
	IVH	IVH	
**Model 2**	PS-CST rFA	-	0.82 (0.68–0.95)
	PS-CST rMD	PS-CST rMD	
	PS-ICH score	ICH score	

*ICH* intracerebral haemorrhage, *mRS* modified Rankin scale, *FA* fractional anisotropy, *MD* mean diffusivity, *PS-CST* pontine segment of corticospinal tract, *IVH* intra-ventricular haemorrhage, *PHE* peri-haematomal oedema, *GCS* Glasgow Coma Scale, *DTI* diffusion tensor imaging, *LASSO* Least absolute shrinkage and selection operator

## Discussion

Our study confirmed the feasibility of using DTI in the acute phase of ICH to extract metrics that can quantify altered microstructure in CST, which is associated with 6-month functional outcome. Predictive performances of DTI metrics alone were comparable to the existing ICH prognostic score (PS-CST rFA 0.71 [95% CI 0.54–0.89] and PS-CST rMD 0.72 [95% CI 0.53–0.92] *versus* ICH score 0.76 [95% CI 0.61–0.91]). This exploratory study suggests that a model with DTI and non-DTI variables might offer an improvement in the prediction of 6-month functional outcome. However, a larger study is required to investigate this further and determine whether any improvement is statistically significant.

Our findings are consistent with a limited number of previous studies [17–20] , in which DTI metrics were also associated with outcome after ICH. However, given the great methodological heterogeneity among the available studies—related to differences in the study cohorts, the time-period between index event and brain MRI, data acquisition and processing methods, anatomical site of DTI measurements, and outcome measures—the potential clinical benefit of the application of DTI in ICH has been difficult to assess.

Only one study has compared the predictive performance of DTI parameters and the ICH score [21] and found that the prognostic value of the ICH score substantially surpassed that of CST-related DTI metrics (AUC 0.74 vs. 0.44; *p* = 0.01 for mRS > 2); moreover, the combination of the DTI metrics with the ICH score did not improve the prognostication of outcome. Our findings appear more promising regarding the potential predictive clinical value of DTI metrics; our estimates suggest that combined models with DTI and non-DTI variables (including ICH score) might provide better outcome discrimination than ICH score alone, although the difference in discrimination was not statistically significant. The discrepancy might be due to differences in population characteristics: 68.8% of patients included in the cited study had lobar ICH, and given their variable location and impact on CST fibres, lobar ICH is likely to have less consistent impact on CST. By contrast, we included only patients with deep supratentorial ICH; as shown in Fig. 3, the ICH location in our cohort included putamen, globus pallidus, internal capsule, thalamus and external capsule. Haemorrhage and peri-haematomal oedema in these regions are expected to consistently affect the CST via both direct disruption or displacement of fibre tracts and indirect mechanisms including oedema, inflammation and early Wallerian degeneration. Our study suggests that in acute ICH, DTI metrics in the pons can detect microstructural alterations of the CST which are associated with 6-month mRS.

The timing of measuring microstructural changes in DTI is of potential importance because Wallerian degeneration of CST following acute insult is a dynamic evolving phenomenon. [22] A recent study [23] on mixed (lobar and deep) ICH found no changes in FA at the cerebral peduncle within 12 h after ICH, which might have been due to the ultra-early timing of MRI acquisition in the study. By contrast, we included patients with MRI performed within 5 days after ICH; in the vast majority of patients (95.2%), MRI was performed at least 24 h after the index event. These findings suggest that DTI metrics might be of greater predictive value if measured beyond the hyperacute phase of ICH. On the other hand, our study demonstrated that DTI parameters are predictive of functional outcome even at an early stage, in which often some important clinical decisions must be made in ICH patients (e.g. in relation to admission to neurocritical care or consideration of neurosurgery).

Our findings suggest that quantitative MRI (DTI) is feasible and of potential clinical predictive value that could add information beyond that from standard neuroimaging in acute ICH. Direct anatomical involvement of the CST due to haematoma mass effect or destruction is associated with functional outcome after ICH [24], which can be detected by standard structural neuroimaging techniques. However, peri-haematomal oedema, inflammation and early Wallerian degeneration could cause functionally relevant injury to white matter fibres beyond the haematoma itself; our data indicate that DTI provides a quantitative measure of CST integrity related to these mechanisms with potential to add valuable prognostic information to that available from standard structural MRI.

The brain MRIs in our study were performed as part of standard clinical acquisition protocols and did not lengthen or reduce tolerability of the examination. Brain MRI already plays a key role in ICH-related diagnostic work-up in practice; it is widely performed to seek evidence of underlying structural causes (e.g. tumours, cavernomas, macrovascular lesions) and is more sensitive than CT for visualizing biomarkers of the cerebral small vessel diseases that cause most ICH (i.e. white matter hyperintensities, cerebral microbleeds, lacunes and perivascular spaces). The potential predictive ability of DTI metrics, in combination with standard clinical and radiological variables, may further consolidate the role of MRI in acute ICH.

The modified Rankin Scale is the most widely functional outcome scale adopted in stroke studies but is highly oriented on motor outcome which is highly dependent on CST integrity. However, the clinical impact of ICH goes far beyond motor outcome, including cognitive deficits and dementia [25, 26]. Similarly, the role of DTI might go beyond the measurement of Wallerian degeneration in CST. Several studies have already adopted a whole brain approach and have evaluated the association between DTI parameters and cognitive functions [27]. The application of a whole-brain DTI approach to predict ICH functional outcomes is thus potentially of interest for future research.

Our study has strengths. Our population is homogeneous with respect to the timing of MRI, site of ICH and standardized DTI region of interest (PS-CST). A single-rater assessment of ICH and PHE lesions reduced variability in the segmentation process. We also applied a standardized automated post-processing protocol. In view of the modest sample size, we used LASSO estimation, a technique that produces better models for prediction in small datasets.

We also acknowledge limitations. The small cohort size limits the precision of our risk estimates with wide 95% CI, but to the best of our knowledge this is the largest study of deep supratentorial ICH in a Western population. We do not have detailed information on the pattern or severity of motor deficit on hospital admission; however, since all participants had deep ICH and were admitted with clinical symptoms suggesting stroke, it is highly likely that a motor deficit was present in the great majority of those included. A potential advantage of our study is that the findings are generalizable to patients regardless of the severity of their initial motor deficit, which can in any case fluctuate markedly in the first few days after ICH. Although MRI studies are routinely performed for patients with ICH and no contraindications in our centre, the requirement for MRI is still likely to have created a selection bias: the study population includes clinically milder ICH with small hematoma volumes (only 2 patients with baseline GCS < 15 and median ICH volume 5.4 ml [IQR 3.0–11.7]). These aspects might limit the generalizability of our findings to patients with more severe ICH. Nevertheless, the AUC we found for the ICH score is similar to those from studies that evaluated 6-month functional outcome in heterogeneous ICH populations (pooled AUC from four studies: 0.78 [95% CI 0.74–0.82])[4], suggesting that our prediction models may be generalizable to other ICH populations. The mRS, although in part related to motor function, is also affected by other neurological impairments not related to the CST (e.g. cognition); thus, evaluation of whole-brain DTI measures in future studies might improve associations with functional outcome.

## Conclusion

In the acute phase after deep supratentorial spontaneous ICH, DTI is feasible as part of routine clinical care; rFA and rMD measured in the normal-appearing pontine segment of the corticospinal tract demonstrated acceptable prognostic ability for 6-month functional outcome. Our findings suggest that DTI measures show promise to improve early prognostication after acute ICH, but further studies in larger cohorts are needed.

## Data Availability

All de-identified participant data requests should be submitted to the corresponding author for consideration by the SIGNaL Steering Committee.
